# Image Fusion for Radiosurgery, Neurosurgery and Hypofractionated Radiotherapy

**DOI:** 10.7759/cureus.252

**Published:** 2015-03-02

**Authors:** Hiroshi K Inoue, Atsushi Nakajima, Hiro Sato, Shin-ei Noda, Jun-ichi Saitoh, Yoshiyuki Suzuki

**Affiliations:** 1 Dept of Neurosurgery and Radiation Oncology, Institute of Neural Organization and Cyber Center, Kanto Neurosurgical Hospital; 2 Cyber Center, Kanto Neurosurgical Hospital; 3 Department of Radiation Oncology, Gunma University Graduate School of Medicine; 4 Department of Radiation Oncology, Fukushima Medical University

**Keywords:** image fusion, radiosurgery, neuro imaging, hypo-fractionated radiotherapy, skull base, arteriovenous malformation, trigeminal neuralgia, Head and neck

## Abstract

Precise target detection is essential for radiosurgery, neurosurgery and hypofractionated radiotherapy because treatment results and complication rates are related to accuracy of the target definition.

In skull base tumors and tumors around the optic pathways, exact anatomical evaluation of cranial nerves are important to avoid adverse effects on these structures close to lesions. Three-dimensional analyses of structures obtained with MR heavy T2-images and image fusion with CT thin-sliced sections are desirable to evaluate fine structures during radiosurgery and microsurgery.

In vascular lesions, angiography is most important for evaluations of whole structures from feeder to drainer, shunt, blood flow and risk factors of bleeding. However, exact sites and surrounding structures in the brain are not shown on angiography. True image fusions of angiography, MR images and CT on axial planes are ideal for precise target definition.

In malignant tumors, especially recurrent head and neck tumors, biologically active areas of recurrent tumors are main targets of radiosurgery. PET scan is useful for quantitative evaluation of recurrences. However, the examination is not always available at the time of radiosurgery. Image fusion of MR diffusion images with CT is always available during radiosurgery and useful for the detection of recurrent lesions. All images are fused and registered on thin sliced CT sections and exactly demarcated targets are planned for treatment.

Follow-up images are also able to register on this CT. Exact target changes, including volume, are possible in this fusion system.

The purpose of this review is to describe the usefulness of image fusion for 1) skull base, 2) vascular, 3) recurrent target detection, and 4) follow-up analyses in radiosurgery, neurosurgery and hypofractionated radiotherapy.

## Introduction and background

Precise target detection is essential for radiosurgery, neurosurgery and hypofractionated radiotherapy because the treatment results and complication rates are related to the accuracy of target (lesion) definition and visualization (volume evaluation) of the surrounding critical anatomy [1].

In skull base tumors and tumors around the optic pathways, exact anatomical evaluation of the cranial nerves is important to avoid any adverse effects on these structures close to lesions. The selection of microsurgical approaches is also influenced by the relationship between the lesions and surrounding important structures, such as perforating arteries, major veins, the hypothalamus, brainstem and cranial nerves. The three-dimensional (3-D) analyses of structures obtained with MR heavy T2-images, MR angiography, MR venography, and image fusion with CT thin-sliced sections, as well as gadolinium (Gd)-enhanced T1-images, are desirable to evaluate fine structures before microsurgery or during radiosurgery [2]. Image fusion of MR images with CT is also able to correct the distortion of MR images [3].

In vascular lesions, especially in arteriovenous malformations (AVMs), angiography is the most important tool for evaluation of the whole structures from feeders to drainers, shunts, blood flow, and risk factors for bleeding. However, the exact sites and surrounding structures in the brain are not shown on angiography. True image fusions of angiography to MR images, including MR angiography, and to thin-sliced axial planes of CT angiography are ideal for precise target definition for radiosurgery [4]. The information obtained from these images is also useful for deciding on the best surgical approach and for determining the indications for intravascular intervention.

In malignant tumors, especially recurrent head and neck tumors, the biologically active areas of recurrent tumors are the main targets of radiosurgery. PET scanning is useful for quantitative evaluation of recurrences and used for image fusion [5-6]. However, the examination is not always available at the time of radiosurgery. In contrast, image fusion of MR diffusion images with CT is always available during radiosurgery and can be useful for the detection of recurrent lesions throughout the whole body. All other images needed are fused and registered on thin-sliced CT sections, and precisely demarcated targets are planned to treat the whole body via radiosurgery, including brain radiosurgery.

Follow-up images are also able to be compared with these CT-based images. Exact target changes, including changes in the tumor volume, marginal recurrences, and obliteration changes of AVMs, are possible to evaluate using this fusion system. Reduction rates after treatment are obtained from measurement of the exact tumor volume after treatment on the same registration system. Diagnosis of marginal recurrences is important for determining the indications for repeat treatment and precise target evaluation for radiosurgery. For AVMs, nidus changes show obliteration patterns after radiosurgery, and may suggest the mechanism by which the radiosurgical effects impact on the vascular structures.

The purpose of this review is to describe the usefulness of image fusion for 1) skull base, 2) vascular, 3) recurrent target detection, and 4) follow-up analyses after radiosurgery, neurosurgery and hypofractionated radiotherapy.

## Review

### Radiological examinations for image fusion

CT scans, MR images of multiple sequences, PET, and angiography are generally used for image fusion. From a practical perspective, image examination methods used in our institute are shown here as examples.

A Fast CT scan with 64x2 rows detectors (SOMATOM Definition AS+, Siemens, Germany) is used to obtain 0.6-1mm sliced axial images for fusion. Then 3-D rotation angiography is performed, and thin-sliced axial images are reconstructed (Axiom Artis, Siemens, Germany). Heavy T2-images (constructive interference steady state (CISS) sequence) of 0.8 mm sliced axial images and diffusion images are obtained using a 3.0T MR machine (Achieva 3.0T Quasar Dual, Philips, Netherlands), which is also used with other modes, such as T1-weighted image, T2-weighted image, proton density, Gd-enhanced T1-image, MR angiography, and MR venography [7]. Magnetization prepared rapid acquisition gradient echo (MPRAGE) or diffusion tensor imaging (DTI) for tractography is also used [3, 8].

**Figure 1:**
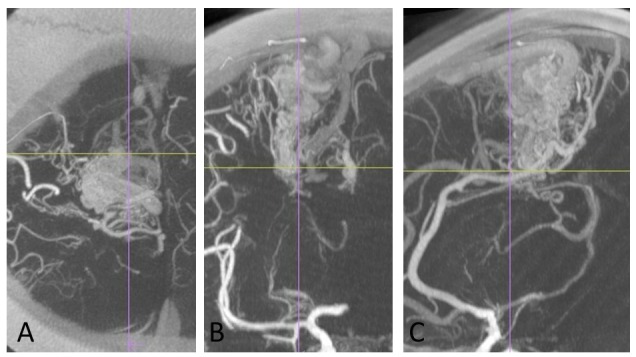
Three-dimensional evaluation of an arteriovenous malformation. The 3-D fine structures of an arteriovenous malformation in the right frontal lobe reconstructed from axial images of rotation angiography with the DICOM image analysis. The feeding arteries, nidus, and draining veins are clearly shown, as the intranidal structures. A: axial, B: coronal, C: sagittal view. The original 1 mm-axial image is used for true image fusion of angiography to thin-sliced CT for radiosurgery.

The 3-D fine structure evaluation by angiography and MR heavy T2-images is performed with the DICOM image analysis software (Ziosoft Inc., Japan), as shown in Figure 1.

**Figure 2:**
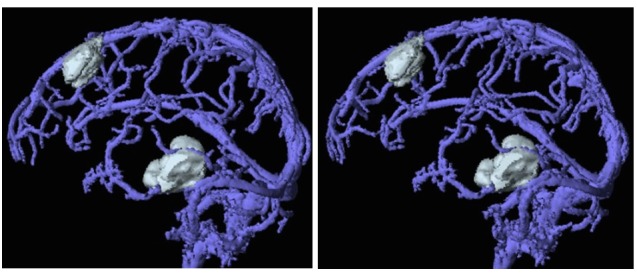
Three-dimensional evaluation of brain tumors and their surrounding veins. A merged image of a Gd-enhanced T1-image and MR venography showing the relationship between tumors and surrounding veins. The stereo view.

Merging of the MR diffusion image and CT data is done with the Work Station software program (Virtual Place Plus, AZE Ltd., Japan). Merging of the view of heavy T2-images and MR angiography or Gd-enhanced T1-images and MR venography examined simultaneously is performed using the Merge View software program (Achieva 3.0T Quasar Dual, Philips, Netherlands) as shown in Figure 2.

The 3-D merged views of heavy T2-images and MR angiography or Gd-enhanced T1-images and MR venography are used for microsurgical approaches and lesion evaluation for radiosurgery/hypofractionated radiotherapy. The merged view of MR diffusion images and contrast-enhanced CT is used to identify recurrent lesions in cancer patients.

### Image fusion

Image fusion can be performed by radiation therapists or physicists, and the automatic image fusion is reliable in most cases. Our methods are described here as practical examples.

**Figure 3:**
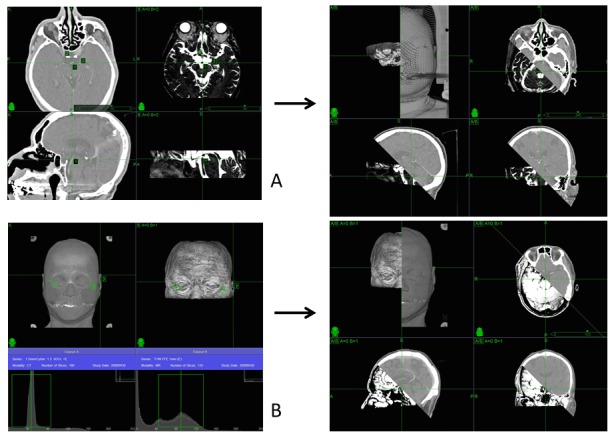
MR to CT image fusion registration. Image fusion of point-based registration (A) and intensity-based registration (B). MR heavy T2-images fused to thin-sliced CT axial sections using point-based and Gd-enhanced MR images using intensity-based registration. Before (left) and after image fusion (right).

Image fusion to CT 0.6-1 mm slices is performed using all transported MR images (Gd-enhanced T1-images, proton density, heavy T2-images, etc.) and angiographic images on the MultiPlan system (Accuray, Inc., Sunnyvale, CA, USA). The methods used for image fusion are 1) point-based registration, 2) intensity-based registration, and 3) manual registration (correction). Point-based registration involves determining the 3-D coordinates of corresponding points in the two images and computing the transformation that best aligns these points. Intensity-based registration involves calculating a transformation between two images using a measure of alignment based only on the values of the pixels or voxels in the images [9]. In practice, three to four anatomical points, such as the union or bifurcation of arteries identical on each CT and MR image, are used in the point-based registration (Figure 3A). Data regarding the body surface are used in intensity-based registration (Figure 3B). Manual correction is used after automatic fusion to adjust target points on all images to these on CT slices.

The image fusion of Gd-enhanced T1-images to CT is usually used for radiosurgery for tumors, and is also useful for neurosurgery. Additional fusion of heavy T2-images to CT is used for skull base lesions for radiosurgery and microsurgery. True image fusion of angiography to axial sections of CT angiography is for vascular lesions, such as AVMs and dural AV fistulas. Additional fusion of Gd-enhanced MR angiography to CT angiography is used to visualize brain structures, especially in patients who received intervention, because embolic materials sometimes disturb normal visualization of CT images.

### Skull base target detection

**Figure 4:**
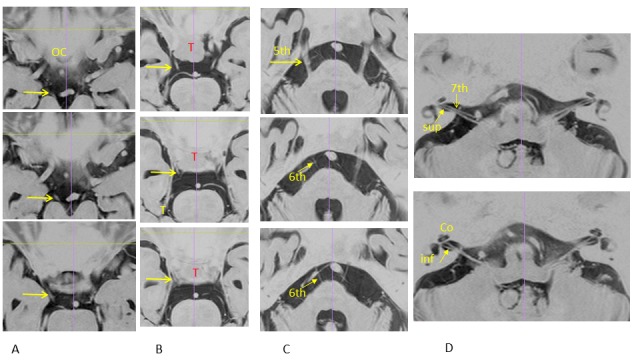
Skull base target detection (microsurgical anatomy). Fine structures of cranial nerves obtained with MR heavy T2-images reconstructed using DICOM image analysis software. A: The optic chiasm (OC) and oculomotor nerve (arrows). B: The oculomotor nerve (arrow) close to a tumor (T). C: The trigeminal nerve (5th) and abducens nerve (6th). D: The facial nerve (7th), superior vestibular (sup) and inferior vestibular (inf) nerves, cochlea (co), and cochlear nerve. (Recommend to use magnified view)

The detailed structures of the cranial nerves, such as the optic pathway, third nerve, three branches of the trigeminal nerve, sixth nerve, facial nerve, cochlear nerve, superior and inferior vestibular nerves, glossopharyngeal nerve, vagal nerve, accessory nerve, and hypoglossal nerve are clearly shown in 3-D images from heavy T2-image analyses at the submillimeter level as microsurgical anatomy (Figure 4).

Image fusion of heavy T2-images to thin-sliced CT is helpful to evaluate the presence and localization of nerves in bony structures and for dose planning of radiosurgery/hypofractionated radiotherapy. Precise detection of these nerves and image fusion on CT are useful to limit the marginal dose of radiosurgery/hypofractionated radiotherapy and is essential to avoid adverse effects on surrounding structures.

1) Lesions Around the Optic Pathway and Oculomotor Nerve

**Figure 5:**
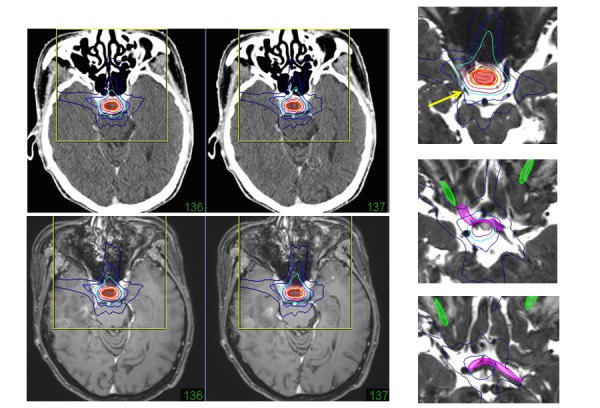
A sellar tumor and the surrounding structures. A Gd-enhanced MR image (lower left) fused to thin-sliced CT (upper left). Image fusion of the heavy T2-image showing the dose plan sparing the oculomotor nerve (yellow arrows), optic nerve (green), and optic chiasm (pink).

Many tumors, such as meningioma, pituitary adenoma, craniopharyngioma, optic glioma, and paranasal sinus carcinoma, originate in and around the optic pathways. Preservation of visual function is important for patients with these tumors. Precise target detection and optic pathway delineation are essential to avoid the adverse effects of radiosurgery/hypofractionated radiotherapy and surgical damage during microsurgery. Displacement, compression, and deformity of the optic pathway is shown on MR heavy T2-images and near bony structures on CT. During dose planning for radiosurgery, sparing the optic pathway (volume evaluation) and oculomotor nerve from treatment dose line is possible in cases using image fusion, as shown in Figure 5.

2) Trigeminal Nerve, Offending Artery, and Abducens Nerve

**Figure 6:**
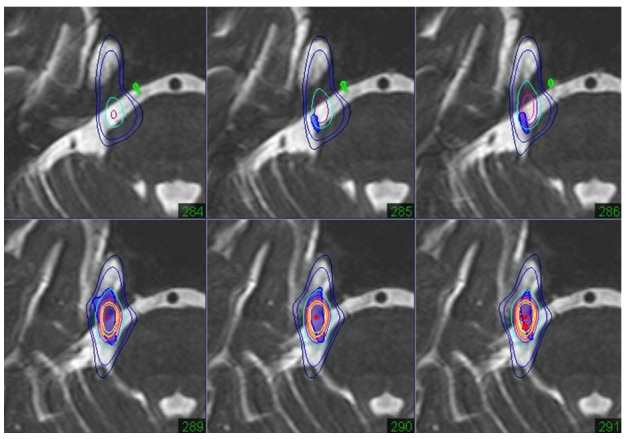
Trigeminal neuralgia. Heavy T2-images (fused to thin-sliced CT) of the trigeminal nerve showing the three branches of the pre-ganglion portion, the Gasserian ganglion, and the retro-ganglion portion. The retro-ganglion portion at the petrous apex is targeted, and the abducens nerve (green) is out of prescribed isodose range.

Idiopathic trigeminal neuralgia is caused by chronic vascular compression of the trigeminal nerve and disappears immediately after decompression of the offending artery with microsurgery. Changes in the trigeminal nerve, such as deformity, angulations, and displacement, are clearly shown in the merge view of MRI and MRA. The merge view is useful for surgical approaches used during microvascular decompression. The 3-D image of the trigeminal nerve is also required for radiosurgery [10]. In dose planning for radiosurgery using image fusion of heavy T2-MR images to thin-sliced CT, the target point is clearly shown and the abducens nerve is out of the prescribed isodose line, as shown in Figure 6.

3) Acoustic Tumors, Facial Nerve, Vestibular Nerve, and Cochlea

**Figure 7:**
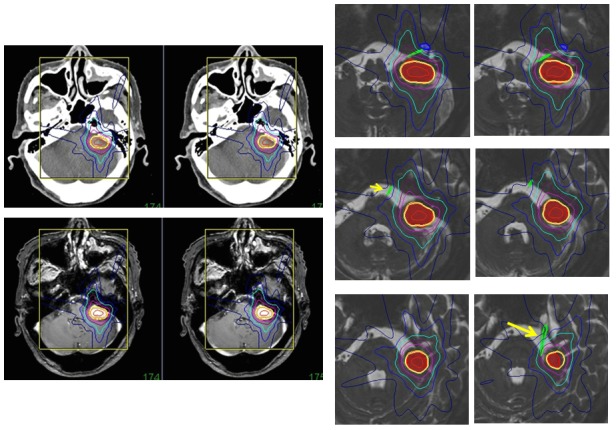
A cerebello-pontine angle tumor and the surrounding structures. A Gd-enhanced MR image (lower left) fused to thin-sliced CT (upper left). The image fusion of a heavy T2-image showing the dose plan sparing the 7th and 8th nerves (green), cochlea (blue), 6th nerve (small yellow arrow), and 5th nerve (large yellow arrow).

In the era of radiosurgery, hearing preservation is an important goal of treatment for acoustic tumors [11]. The 3-D heavy T2-imaging can clearly show the superior and inferior vestibular nerves, facial nerve, cochlear nerve, cochlea, and semicircular canals (see Figure 4). The facial nerve around small acoustic tumors is also usually shown on the ventral side of tumors. The extent of the intracanalicular part of the tumor is then shown by MR and CT image fusion. In dose planning for radiosurgery/hypofractionated radiotherapy, the isodose on these neural structures should be limited for functional preservation (Figure 7).

4) Jugular Foramen Tumor, 9-11th Nerves, and Hypoglossal Nerve

**Figure 8:**
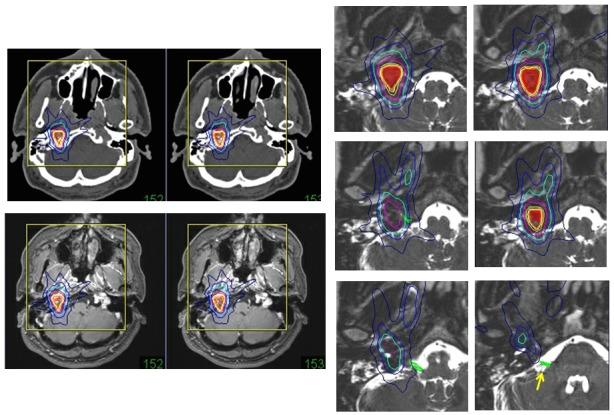
A jugular foramen tumor and the surrounding structures. A Gd-enhanced MR image (lower left) fused to thin-sliced CT (upper left). The image fusion of a heavy T2-image showing the dose plan sparing the 9th-11th nerves (green) and 8th nerve (green with a yellow arrow).

Patients with jugular foramen tumors have minor or major symptoms of the lower cranial nerves. Preservation of swallowing function is important for such patients [12]. Image fusion of heavy T2-images to thin-slice CT is useful to detect the cranial nerves and to spare them during the dose planning of radiosurgery/hypofractionated radiotherapy, as shown in Figure 8.

### Vascular target detection

Digital subtraction angiography is essential for evaluation of vascular lesions, such as AVMs and fistulas. Information about the feeding arteries, blood flow, intranidal aneurysms, varices, and draining veins is important in order to understand the bleeding risk and indications for intervention prior to microsurgery and radiosurgery. MR imaging is also important for evaluation of the surrounding structures to avoid complications. True image fusion, but not the overlay of angiography to MR images and CT, is ideal for radiosurgery to detect the precise target and to spare functional structures [4, 13].

1) Brain Arteriovenous Malformation

**Figure 9:**
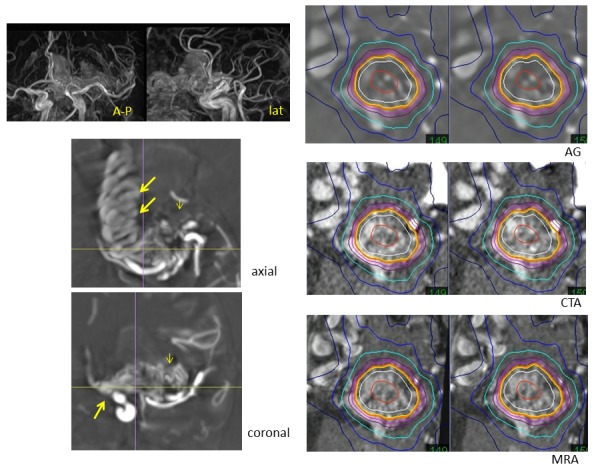
A brain arteriovenous malformation. MR angiography showing a large AVM in the left frontal lobe (upper left). The 3-D reconstruction from axial images of rotation angiography (lower left) showing the nidus (small arrow) and massive draining veins (large arrows) in the frontal base. Dose plan targeting of the nidus, excluding massive drainers, on axial images of the angiogram (AG) fused to those of CT angiogram (CTA) and enhanced MR angiogram (MRA).

Total obliteration without functional deficits is the goal of AVM treatment [14]. The nidus delineation, excluding feeder and draining veins, is important during dose planning for radiosurgery. The 3-D vascular structures of AVMs are reconstructed to detect the nidus from rotation angiography (Figure 1). Intranidal fine pathologies, such as arteriovenous shunts, small aneurysms, and varices, can be evaluated even after intervention. True image fusion angiography to CT (axial slice CT angiography) and MR images, including contrast-enhanced MR angiography, enables the detection of tiny pathologies and complicated targets after intravascular intervention. These targets are difficult to detect with conventional methods using stereotactic multidirectional angiography. Exact nidus detection is important for radiosurgery to avoid complications due to overdoses to the surrounding brain, especially in cases of large AVMs (Figure 9).

2) Spinal Arteriovenous Malformation

**Figure 10:**
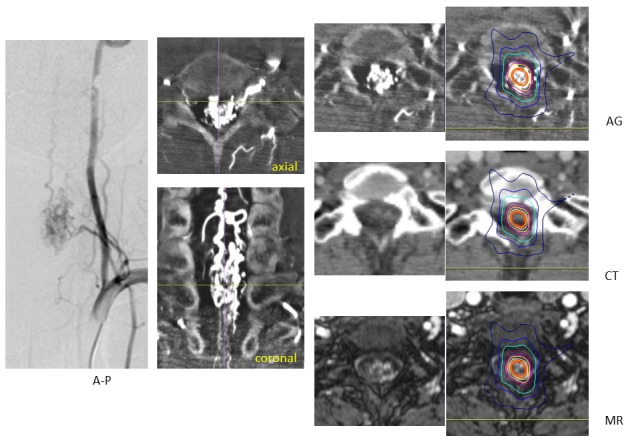
A spinal arteriovenous malformation. Digital subtraction spinal angiography showing an intramedullary AVM in the cervical spinal cord (left). The 3-D reconstruction from the axial images of rotation angiography (middle line) showing the nidus and draining veins. The dose plan targeting the nidus while sparing the surrounding spinal cord, with the steepness of the isodose line on axial images of an angiogram (AG) fused to CT and enhanced MR images (right line).

Spinal AVM is one of most difficult vascular diseases to treat in neurosurgery. Patients with spinal AVMs, especially intramedullary AVMs, have risks of tetraparesis or paraparesis due to hemorrhage or surgical procedures. Whole body (spinal) radiosurgery is an option to treat spinal AVMs with low treatment risks. Precise target detection is essential for radiosurgery, and true image fusion of spinal angiography is desirable for dose planning (Figure 10). Detailed nidus evaluation and sparing of the surrounding spinal cord are required to avoid adverse effects on functional spinal tracts.

3) Dural Arteriovenous Fistula

**Figure 11:**
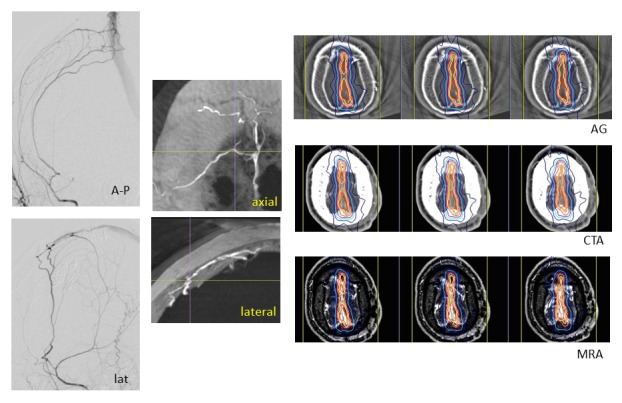
A dural arteriovenous fistula (AVF). Digital subtraction angiography showing a dural AVF of the superior sagittal sinus (left). The 3-D reconstruction from axial images of rotation angiography (middle line) showing fistulas to the sinus. The fistula points are clearly shown in 3-D images (crossed line). The dose plan targeting the AVF and the sinus wall on axial images of an angiogram (AG) fused to those of CT angiogram (CTA) and enhanced MR angiogram (MRA).

Dural AVFs are usually indications for intervention, and transvenous approaches are most frequently used. The 3-D reconstruction images from rotation angiography are useful for intervention and direct surgery. When patients have recurrent lesions after intervention, risks of thrombosis of a major venous sinus, or have medical contraindications for surgical procedures, radiosurgery is a less invasive and safer treatment for dural AVFs. Total obliteration preserving major venous sinuses is obtained more safely and earlier than brain AVM obliteration after radiosurgery [15-16]. True image fusion of angiography via CT and MR images is useful to detect fistula points and to determine the targets for radiosurgery, as shown in Figure 11.

### Recurrent target detection

**Figure 12:**
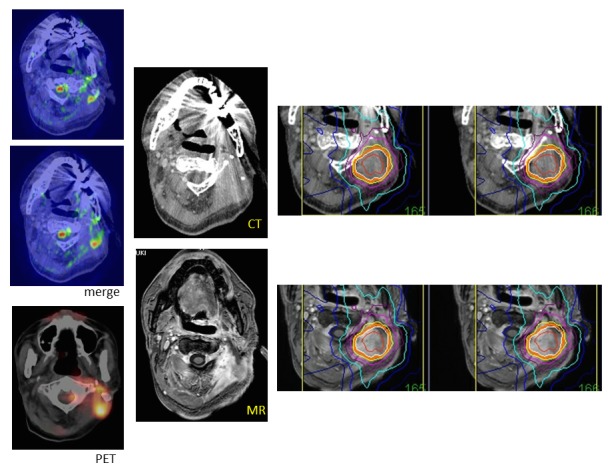
A recurrent neck cancer. MR diffusion images merged to thin-sliced CT axial sections showing a recurrent lesion in the neck, in the same locations as indicated by the PET scan (left line). The Gd-enhanced MR image is fused to CT and used for dose planning (center). The target of hypofractionated radiotherapy is demarcated based on the merged images.

PET scanning is usually used to detect recurrences and metastases in follow-up studies of patients with cancers. MR diffusion imaging is also helpful to detect recurrent lesions during radiosurgery, especially recurrent head and neck tumors, as shown in Figure 12. However, the exact site of positive areas is not clearly shown. Image fusion with CT and MR diffusion images will enable the delineation of the target for radiosurgery, as well as PET scanning [17-18]. Direct image fusion of MR diffusion images to thin-sliced CT for radiosurgery is not precise yet. At present, merged images of MR and CT are made, and the target is delineated on the merged images (Figure 12). Then, image fusion of Gd-enhanced MR images to CT is performed for dose planning prior to radiosurgery. The image fusion supported with merged images is useful, especially for patients who received reconstructive surgery after removal of head and neck tumors, because the normal structures are often not shown, and recurrent lesions are difficult to find using CT or MR imaging without the accompanying information provided by PET scans or MR diffusion images.

### Follow-up analyses

In follow-up examinations, the quantitative analyses are important to detect fine changes after treatment [19]. MR images are usually used for follow-up studies of brain lesions. However, the examination angle is not identical between studies. Image fusion using registered CT during radiosurgery can be very useful to calculate volume reduction rates, to decide on the target of recurrent lesions, and to evaluate fine structural changes after treatment.

1) Measurement of Reduction Rates

**Figure 13:**
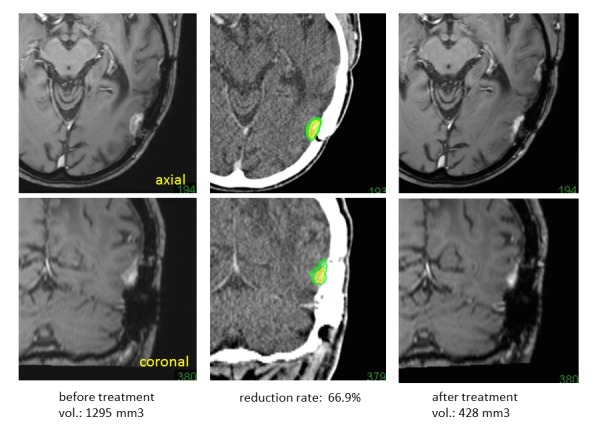
Measurement of tumor reduction rates. MR images fused to thin-sliced CT before radiosurgery (left). Follow-up MR images after treatment (right) also fused to CT (base on images taken during radiosurgey). Both images are able to compare with identical axial (upper line) and coronal (lower line) images using image fusion. The quantitative analysis (of the reduction rate) is also possible from the volume measurements taken before and after treatment (center).

Similarly, the quantitative analyses after treatment are used to evaluate the sensitivities for treatment and dose responses after treatment, etc. However, radiological examination using MR imaging is not always performed with the same machine and with patients in the same position, even when imaging is performed in the same hospital. Image fusion of such follow-up MR images to registered CT for radiosurgery enables the changes to be compared after treatment on the same axial images with identical angles. To accurately measure lesion volumes after treatment, the exact volume changes and reduction rates are available, as shown in Figure 13, even for small tumors. The rate is calculated as (volume before treatment – volume after treatment)/volume before treatment.

2) Marginal Recurrences

Retreatment after radiosurgery is infrequently required for recurrent tumors, such as brain metastases, gliomas, and malignant meningiomas. Radiation necrosis is the most severe adverse effect of radiosurgery, and the risk increases based on the volume during retreatment and is more severe than in the first treatment. The retreatment volume should therefore be minimized, excluding the controlled part after the primary treatment. Exact evaluation of recurrent lesions and detection of marginal recurrence is essential for retreatment of radiosurgery. Image fusion of follow-up MR images is useful for the meticulous analyses of changes after treatment, especially for marginal recurrences.

**Figure 14:**
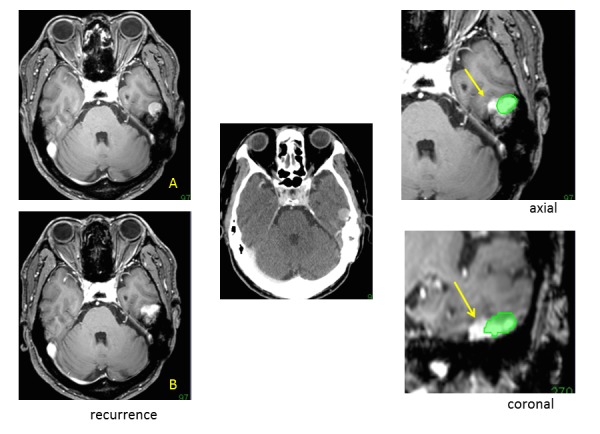
Detection of marginal recurrence. A follow-up MR image after treatment showing recurrence (B) compared with the image before treatment (A). Image fusion using thin-sliced CT (based on the image from radiosurgery, center) enabled us to detect the treated part of the tumor (green) and the recurrent tumor from the medial margin of the tumor (arrows) on identical axial and coronal images (right). The treated part decreased in volume, as is shown in follow-up MR images fused with the image before radiosurgery.

Evaluation and targeting of marginal recurrence, excluding the controlled part, is helpful to avoid the adverse effects of retreatment for recurrence after radiosurgery. Differential detection of the controlled part and marginal recurrence can be easily detected with the image fusion analyses as shown in Figure 14.

3) Obliteration of AVM

**Figure 15:**
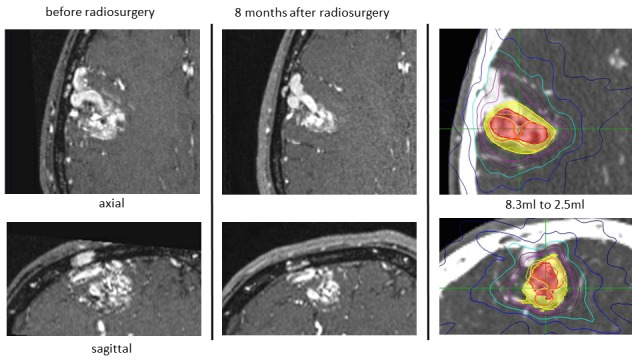
Follow-up evaluation of an arteriovenous malformation. Follow-up MR images of an arteriovenous malformation after treatment (center) fused to thin-sliced CT (baseline image) could be compared with images taken before radiosurgery (left) with identical axial, coronal, and sagittal images. Not only the reduction rate (right: yellow showing the size before treatment, and red showing the size after treatment) but also fine changes of intranidal structures, feeding arteries, and draining veins could be seen.

Evaluation of vascular changes after radiosurgery is necessary for follow-up studies of AVM. Volume reduction, feeder size reduction, structural changes of the nidus, and drainer size reduction are now clearly identifiable in enhanced MR angiography without conventional angiography [20]. Image fusion of follow-up MR images to registered CT is useful to obtain more information about the obliteration of the nidus and the obliterated part in 3-D images (Figure 15).

Sequential follow-up studies using the image fusion system show fine changes of the nidus structures, and the meticulous analyses may suggest the mechanism responsible for the vascular obliteration after radiosurgery.

## Conclusions

Image fusion is useful to detect skull base, vascular, and recurrent targets that are difficult to delineate in usual examinations of MR and CT images. The fusion of multiple images on CT is important for radiosurgery, neurosurgery and hypofractionated radiotherapy to increase the efficacy of treatment and to avoid complications. Follow-up examination using image fusion is also helpful for the meticulous analyses of reduction rates after treatment. Evaluation of marginal recurrences decreases the incidence of adverse effects related to retreatment. In addition, sequential studies of vascular obliteration may suggest mechanisms responsible for the radiosurgical effects on AVMs, allowing for the development of more effective treatments.
